# Effects of Light on Attention and Reaction Time: A Systematic Review

**DOI:** 10.34172/jrhs.2021.66

**Published:** 2021-10-31

**Authors:** Rostam Golmohammadi, Hanieh Yousefi, Negar Safarpour Khotbesara, Abbas Nasrolahi, Nematullah Kurd

**Affiliations:** ^1^Department of Occupational Health and Safety Engineering, School of Public Health, Hamadan University of Medical Sciences, Hamadan, Iran; ^2^Department of Ergonomics, School of Public Health, Hamadan University of Medical Sciences, Hamadan, Iran; ^3^Research Center for Prevention of Psychosocial Injuries, Ilam University of medical science, Ilam, Iran

**Keywords:** Light, Lighting, Attention, Refractory Period

## Abstract

**Background:** Accuracy, speed, efficiency, and applicability of activities in the workplace are among the most important effective factors on people's productivity, which is in turn affected by environmental factors, such as light. Therefore, the present research aimed to review the studies performed about the effects of light on attention and reaction time.

**Sudy Design:** A systematic review.

**Methods:** This review study systematically searched articles from 2000-2019 in databases of Google Scholar, ISC, SID, Magiran, Web of Science, Science Direct, PubMed, and Scopus using keywords of light, lighting, attention, and reaction time. The titles and abstracts of articles containing relevant results over the past 20 years were extracted. Thereafter, they were categorized and analyzed according to the title, author name, publication year, study method, study type, and evaluation results.

**Results:** Based on the results, the light with shorter wavelengths, higher intensity, and higher color temperature led to suppressed melatonin, higher consciousness, less somnolence, increased attention, and faster reaction time. Simultaneous exposure to harmful levels of environmental factors affects cognitive and physiological parameters, acting independently with a separate mechanism or synergistically with a similar mechanism. The best light in the regulation of psychological, biological, and cognitive processes is bright daylight in the morning with a short wavelength, high intensity, and more lasting effects.

**Conclusion:** As evidenced by the obtained results, light is a powerful modulator of non-visual performance in cognitive tasks. The wavelength, color temperature, and light intensity modulate brain responses to cognitive tasks, including attention and reaction time. Therefore, these parameters, along with personal and environmental factors, should be considered in designing and using light.

## Introduction

Today, modern technologies have changed the working environment, creating more visual and cognitive needs than just physicalones^1^. Based on the studies conducted in recent decades, good illumination conditions modulate human needs regarding working, economic, environmental, and design-architectural requirements. Human performance, apparent space, safety, health, and well-being are improved by taking advantage of good lighting conditions^2^. Accuracy, speed, efficiency, and applicability of activities in the workplace are among the most effective factors on people's productivity, which is in turn affected by environmental factors, such as light^3^. The human visual system does not work optimally in poor lighting which leads to information loss, increased errors, and decreased performance ^4^.

Numerous studies have demonstrated that proper lighting exerts a positive impact on work performance, reducing accident rates. Moreover, inadequate lighting increases eye strain, reduces performance, and leads to accidents^4-6^. Human factor research on lighting has largely focused on light visual aspects, as well as visual disturbance and performance. Evidence on the non-visual, psychological, and biological effects of light has recently been presented^7^.They revealed that different lighting conditions significantly affect many non-visual functions, such as physiological and psychological mechanisms, and biological-cognitive processes, such as Circadian Rhythm, consciousness, core body temperature, hormone secretion, and sleep^8-10^. Furthermore, several laboratory studies have pointed out that exposure to higher levels of illumination leads to lower melatonin secretion, increased physiological arousal, higher consciousness, as well as improved continuous attention and cognitive function^11,12^.

Attention and reaction time are among the important human cognitive indices. Attention is a cognitive process defined as a selective focus on one aspect of the environment while ignoring others^13^. The word "attention" can be defined in accordance with the number of errors made during a test. Accordingly, more careful attention during the test leads to fewer errors and vice versa. Furthermore, there is a close relationship between attention and reaction time^14^. That is to say, the higher levels of attention result in a shorter reaction time, and the opposite is also true. Reaction time is the time elapsed between understanding a situation and the response provided by an individual ^15^. In humans, it may last from 0.5-> 3 sec, depending on the type of activity, attention, and consciousness^16,17^.

 Some studies assessed the effect of lighting on cognitive functions; nonetheless, they have not reached a clear and definite conclusion 18. According to the aforementioned issues, although various studies have been conducted on cognitive functions and their importance, there is a dearth of research pertaining to the effect of inappropriate lighting on cognitive functions, including attention and reaction time. Attention and reaction time have a significant role to play in human errors and the occurrence of accidents; therefore, it is highly important to analyze the influential factors affecting them in the workplace.

## Methods

**Figure 1:**
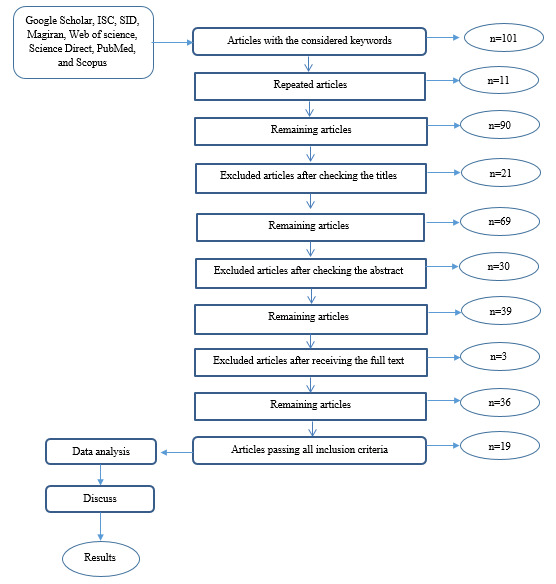
Flowchart of the literature review

This review study investigated the effects of lighting on attention and reaction time. To collect the required data, a query was conducted on six available electronic databases, including Google Scholar, ISC, SID, Magiran, Web of Science, Science Direct, PubMed, and Scopus. The search was performed using the keywords of light, lighting, attention, and reaction times. At each stage, the searched articles in each database were entered into the endnote software. In the first stage, a total of 187 documents related to the topic were entered into the software. In the second stage, according to the framework selected for the study based on a review of published studies from 2000-2019, the relevant documents before this period were deleted, yielding 101 articles. Since many of the records found in various databases were indexed, duplicates were inevitable. Therefore, in the third stage, the duplicates were removed; as a result, 90 documents remained for review. In the next step, the titles of these articles were carefully reviewed, and 21 irrelevant ones were deleted. After the revision of their abstract, another 30 documents were excluded from the assessment due to their irrelevant methodology. Following that, the complete file of the remaining articles was received; however, the full text of three articles could not be accessed, and they were excluded from the review process. The examination of the full texts revealed that 11 articles were not closely related to the subject in terms of purpose, method, and results; therefore, they were ruled out. A diagram of the study selection process is displayed in Figure 1.

 For performing the study, one of the researchers was assigned to review the literature and check for the inclusion and exclusion criteria based on the title and abstract. After the removal of the articles that failed to pass the inclusion criteria, the full text of all the selected ones was investigated. Thereafter, the desired results were extracted considering a number of focused parameters and handed over to another researcher to review and revise them, if necessary. In general, out of 101 documents, 19 articles were investigated to extract the results.

In this study, independent variables included light characteristics (e.g., intensity, wavelength, and color temperature), environmental factor (e.g., noise, heat, and vibration), and personal factors (e.g., gender, sensitivity, and duration exposure), while attention and reaction time were regarded as dependent variables. Each of the reviewed articles examined several variables; therefore, the results of the reviewed articles were classified and evaluated in three main areas: (1) the effects of light-related factors on attention and reaction time (Table 1); (2) the combined effects of light and other environmental factors on attention and reaction time (Table 2); and (3) the effects of personal factors related to light sensitivity on attention and reaction time (Table 3). Moreover, the effects of daylight and other comprehensive studies on cognitive processes, including attention and reaction time, were investigated.

**Table 1:** Articles related to the effects of light factors on attention and reaction time

**Line**	**Title**	**Authors**	**Study method**	**Study type**	**Results**
1	Effects of bright and blue light on acoustic reaction time and maximum handgrip strength in male athletes: a randomized controlled trial^29^	Knaier et al. 2017	A number of 74 male athletes were randomly allocated to bright light (BRIGHT), monochromatic blue light (BLUE), or a control condition (CONTROL). Light exposure lasted for 60 min and started 17 h after the individual midpoint of sleep.	Experimental	Bright light might reduce melatonin levels; nonetheless, neither bright nor blue light exposure in the evening seems to improve reaction time or handgrip strength in athletes.
2	A higher illuminance induces alertness even during office hours: Findings on subjective measures, task performance, and heart rate measures^11^	Smolders et al. 2012	The study employed a mixed-group design (n=32), testing effects of two illuminance levels (200 lx or 1000 lx at eye level, 4000 K) during one hour of morning versus afternoon exposure	Experimental	Effects of illuminance on subjective alertness and vitality, sustained attention in tasks, heart rate, and heart rate variability
3	Effects of blue light on cognitive performance^30^	Bansal et al. 2017	During the 5-hour daytime study, seven healthy male participants were exposed to two different screen interfaces for three and half hours (3:30) under a controlled environment.	Experimental	Continuous exposure to LED screens brought about a decrease in frontal region delta theta activity and increased alertness.
4	Effectiveness of classroom lighting colors in students’ attention and meditation extracted from brainwaves^37^	Bozkurt et al. 2014	Attention and meditation levels are extracted from the observed brainwaves of randomly selected two students when changing classroom lighting colors.	Experimental	The attention level of subjects was higher in the red lightning color of the classroom, in comparison with white and green colors.
5	Bright light effects on working memory, sustained attention, and concentration of elderly night shift workers^33^	Kretschmer et al. 2012	A group of four subjects took various visual cognitive performance tests simultaneously in a closed 5.2m-7.4m-3.8m room with a rectangular base area.	Experimental	Bright light at night reduced error rates for a working memory task and a concentration performance task.
6	Effects of correlated color temperature on focused and sustained attention under white LED Desk lighting^40^	Huang et al. 2014	Three correlated color temperature conditions (2700, 4300, and 6500 K) were examined, and the Chu Attention Test was used to measure focused and sustained attention.	Experimental	Correlated color temperatures affected attention. In specific, the 4300 K condition resulted in significantly better focused and sustained attention.
7	Evaluation of the effects of different levels of lighting on attention and reaction time under laboratory conditions^59^	Dehghan et al. 2017	A number of 33 subjects (age range of 19-26 years) underwent cognitive tests. Participants were exposed to three levels of lighting (200, 500, and 1500lux) in laboratory conditions while performing continuous performance tests.	Experimental	Significant differences were observed in the percentage of attention and reaction time in different levels of lighting.
8	The relationship of sleep quality and mental fatigue in different levels of lighting on attention and reaction time in thermal comfort condition^67^	Mohebian et al. 2016	The participants were exposed to 500, 200, and 1500 lx lightings for 1.5 h at a temperature of 22Cº to take the continuous performance test, the reaction time test, the Pittsburgh Sleep Quality Inventory (PSQI), and the mental fatigue checklist.	Experimental	The relationship of sleep quality and mental fatigue before and after exposure to different levels of lighting with the reaction time was significant.
9	Luminance contrast has little influence on the spread of object-based attention68	Watson et al. 2013	The interaction between selective attention and luminance contrast with a contour-grouping task that provides a sensitive measure of the spread of object-based attention was investigated.	Experimental	The spread of object-based attention was largely independent of contrast.
10	The effect of blue-enriched white light on cognitive performances and sleepiness of night-shift workers: A field study^26^	Motamed adeh et al. 2017	Participants were exposed to two lighting conditions for a week. Subjects performed the Conners' Continuous Performance Test II (CPT-II) and 1- back test. A melatonin assessment was carried out.	Before-after interventional	Compared to normal lighting conditions, omission errors and the reaction time during the sustained attention task decreased.
11	Effects of blue and red-enriched light on attention and sleep in typically developing adolescents^28^	Studer et al. 2018	The adolescents participated in two lab days, which took place one week apart. A lab day consisted of two sessions in the light lab, one in the morning, and one in the evening, each lasting for about 1.5 h.	Cross-over	Beneficial medium to large effects of blue-enriched light on attentional performance in two out of three was tasks observed.

**Table 2:** Articles related to combined effects of light and environmental factors on attention and reaction time

**Line**	**Title**	**Authors**	**Study method**	**Study type**	**Results**
1	Effects of combined exposure to noise, heat, and lighting on cognitive performance^42^	Amiri et al. 2015	A number of 128 subjects within the age range of 24-18 years were selected from among the students of Shiraz University of Medical Sciences. The Tool to evaluate cognitive function in this study was Paced Auditory Serial Addition Test.	Experimental	The mean score of attention and working memory were reduced with worsening combined conditions.
2	Investigating the combined effects of heat and lighting on students’ reaction time in laboratory conditions^20^	Mohebian et al. 2016	The study was conducted on 33 healthy students in a thermal stress chamber. The reaction time was measured by a reaction time measurement device after exposure to different heat surfaces and lighting surfaces.	Experimental	Reaction times and reaction time error increased after combined exposure to heat and lighting.
3	Effects of noise, heat, and indoor lighting on cognitive performance and self-reported affect^46^	Hygge et al. 2001	A factorial between-subject design was employed with three independent variables: Noise (38 and 58 dBA), Heat (21 and 278C), and Illuminance (300 and 1500 lx).	Experimental	Interactions were found between noise and heat on the long-term recall of a text and between noise and light on the free recall of emotionally toned words.
4	Combined effect of whole-body vibration and ambient lighting on human discomfort, heart rate, and reaction time^47^	Monazzam et al. 2018	Participants were subjected to 12 experimental steps, each lasting 5 min for four different vibration accelerations in X, Y, and Z axes at a fixed frequency. Three different lighting intensities of 50, 500, and 1000 lx were also considered.	Experimental	The combined effect of vibration and lighting had no significant effect on any of the discomfort, heart rate, and reaction time variables.
5	Evaluation of the Combined Effects of Heat and Lighting on the Level of Attention and Reaction Time: Climate Chamber Experiments in Iran^69^	Mohebian et al. 2018	Study was conducted on 33 healthy students (17 M/16 F). The attention and reaction time tests were performed by continuous performance test and the RT meter, respectively, in different exposure conditions.	Empirical	An increase in heat and lighting level caused a decrease in average attention percentage and correct responses, as well as an increase in commission error, omission error, and response time.

**Table 3:** Articles related to effects of personal factors related to light sensitivity on attention and reaction time.

**Line**	**Title**	**Authors**	**Study method**	**Study type**	**Results**
1	Gender differences in light sensitivity impact on brightness perception, vigilant attention, and sleep in humans^55^	Chellappa et al. 2017	Potential gender differences to evening light exposure of 40 lx at 6500 K (blue-enriched) or at 2500 K (non-blue-enriched), and their impact on brightness perception, vigilant attention, and sleep physiology were investigated.	Experimental	In contrast to women, men showed a stronger response to blue-enriched light in the late evening even at very low light levels.
2	Circadian and gender differences after acute high-altitude exposure: Are early acclimation responses improved by blue light?^74 ^	Silva et al. 2015	A number of 57 volunteers were randomly assigned to two groups: nocturnal (2200-0230 h) or diurnal (0900-1330 h) and exposed to acute hypoxia (4000 m simulated altitude) in a hypobaric chamber.	Experimental	Some tendencies toward better cognitive performance (d2 attention test) were observed under blue illumination.
3	Light effects on behavioral Performance Depend on the Individual State of Vigilance^64^	Correa et al. 2016	Hypothesis was tested by measuring the participants' behavioral state of vigilance before light exposure using the Psychomotor Vigilance Task.	Experimental	Participants with higher levels of basal vigilance before light exposure benefited most from blue-enriched lighting, responded faster in the Sustained Attention to Response Task.

## Results

The selected articles were published in Persian (n=5) and English (n=10) journals. They were generally experimental studies and encompassed various aspects of light and its effects on cognitive functions, including reaction time and attention. The list of final articles which met all the study criteria is presented in Tables 1, 2,3.

## Discussion

In the process of human-machine perception, cognitive activities, such as reaction time and attention, are considered structural elements and main cognitive reactions to external stimuli in order to understand and analyze the conditions of assigned task ^19,20^. Human factor research on lighting has largely on light visual aspects, as well as visual disturbance and performance. Evidence on the light non-visual, psychological, and biological effects has recently been presented^7^. According to various studies reviewed in the present research, the effects of lighting on attention and reaction time can be analyzed as the following:

For indoor lighting, illuminance is one of the important factors which can indicate the quality of lighting conditions. In their study, Yang et al^21^ indicated that illuminance significantly affected subjects’ attention and alertness (P<0.05). That is to say, higher illuminance leads to higher levels of alertness and attentiveness. The participants were most alert, least relaxed, and performed most concentrated under lighting conditions of 500lx. Another laboratory study illustrated that even in the absence of sleep and light deprivation, exposure to a higher illuminance at the eye level can induce subjective alertness and vitality, increase physiological arousal, and improve performance on a sustained attention task^22^. The same results were reported by Leichfried et al who concluded that early morning illumination improves subjective alertness and mood; nonetheless, it had no impact on melatonin level and mental performance of individuals^23^. Generally speaking, a high illuminance level could make subjects feel more alert and concentrated, pointing to its significance in the enhancement of people’s attention level.

Numerous studies have pointed to the positive effects of blue light on function and consciousness^24,25^. For instance, Motamedzadeh et al^26^ demonstrated that compared to baseline conditions and 6500 K, blue-enriched white light (17000 K) effectively improved working memory and sustained attention of control room staff. The performance results of the participants in tasks requiring sustained attention in the mentioned study have also indicated that exposure to blue-enriched white light does not affect the error of action. Nevertheless, compared to baseline conditions, such exposure significantly reduced the number of deletions and response errors. Moreover, the results observed a significant difference between the mean removal error at 17000 K in blue-enriched white light and baseline conditions (*P*=0.020).

In a laboratory study, Baek and Min^27^ showed that exposure to blue-enriched white light after lunch reduced alpha-band activity and improved sustained attention. In the same context, Studer et al.^28^ reported that participants demonstrated increased attention in two of the three attention-based tasks due to blue-enriched light, compared to red-enriched light in the morning (high illuminance about 1000 Lux and short duration less than 1 h). The results also indicated a reduction in reaction time in the performed tests. Along the same lines, Knaier et al^29^ have found that differences in reaction time in the control group (compared to other participants) were 1 and 2 milliseconds (95% CI-9.5) for participants in white light and blue light conditions, respectively.

Bansal et al^30^ also detected significant differences in screened factors, EEG delta/theta activity, mood, sustained attention (reaction time tasks), short-term memory (verbal memory task), and working memory (visual memory task) due to exposure to blue-enriched white light. The effects of blue-enriched white light on the improvement of cognitive function have also been reported in other studies, including Cajochen et al.^31^, Viola et al^24^, Vetter et al^32^, Kretschmer et al^33^, and Chellappa et al.^34^. Nonetheless, previous studies have proved that blue-enriched white light may lead to retinal damage and oxidative stress. Therefore, in order to reduce the negative effects of blue-enriched white light, its direct exposure is prevented^35,36^. Bozkurt et al^37^, in their study on the level of attention in two students with the lighting color change in the classroom, have found that the attention level in both subjects was higher in red light, as compared to that in white and green lights.

Some studies have addressed the effects of correlated color temperature (CCT) on visual function or its physiological and psychological effects^38^. Chellappa et al^39^ have observed that fluorescent lamps with higher color temperatures could enhance consciousness, well-being, and visual comfort. Moreover, exposure to a color temperature of 2700 K led to a faster reaction time in tasks requiring sustained attention, as compared to a color temperature of 6500 K. Their results further have revealed that higher color temperature requires proportional attention in tasks due to its more melatonin suppression and faster reaction time.

Huang et al^40^ have pointed to the effects of color temperature on focused and sustained attention under white LED desk lighting. In the stated study, three CCTs conditions (2700, 4300, and 6500 K) were evaluated, and the Chu attention test was used to measure focused and stable attention. A paired comparison of CCT conditions suggested that at 4300 K color temperature, the score was higher than other conditions. In other words, the focused and stable attention is higher at the mentioned color temperature. The abovementioned results are consistent with those reported by Yamagishi et al^41^ who pointed to the effects of CCT conditions (8200, 5000, 5000, 2500 K: controlled lighting in 470lux) on young and old people using the NV (Night vision) test.

Studies in recent decades have assessed the effect of environmental factors on human comfort, health, and function. However, almost all of them considered the effect of only one factor, and there is a dearth of research on the combined effects of parameters in real conditions. Mohebbian et al.^20^ have pointed out that in thermal comfort conditions (temperature 22°C), the increase of illuminance decreased reaction time and its error, indicating its positive effect on the reaction time. On the other hand, the results of the referred study demonstrated that increasing the temperature (37°C) and illuminance through increasing the reaction time and reaction time error of individuals can interfere with the cognitive process and reduce their performance.

Amiri et al^42^ have indicated that although sound, heat, and light have no negative impact on physiological and cognitive function at their harmless and permissible levels, simultaneous exposure to their harmful levels in different combined conditions exerts a mutual impact on physiological and cognitive parameters (working memory and attention), acting independently with a separate mechanism or synergistically with a similar mechanism^42^. The interaction between light and heat, as well as their impact on cognitive functions, has also been shown in the study by Lucas et al^43^. Huang and Bavolar have also found that the combination of different environmental factors reduces performance. In other words, if their combined effects are more than those of their individual effect, they can intensify each other’s effects and have a similar effect mechanism; however, if the combined effects of environmental risk factors are equal to those of their individual effect, they will most likely have a separate effect^44,45^.

Hygge et al^46^ have demonstrated that tasks were performed more rapidly but less accurately in the presence of the sound component. The stated study observed the significant association of sound, heat, and light with text reading and word recall. These interactions are indicating the theoretical possibility that sound, heat, and internal light directly affect cognitive processes without general or partial mediation, at least not in the way that the inverse U hypothesis suggests. In the same context, Monazzam et al^47^ have observed that an increase in vibration acceleration significantly improved discomfort and heart rate; however, it did not affect the reaction time. The results of the referred study also suggested that vibration and illuminance did not have a significant combined effect on the variables of discomfort, heart rate, and reaction time.

Interpersonal sensitivity to light may affect consciousness, cognitive function, and sleep physiology differently^48^. The impact of light on a wide range of electrophysiological factors may vary from person to person. It has been specified that the function of the visual system is strongly influenced by gender differences^49,50^. Chellappa et al^51^ have assessed the effect of gender differences on light perception, conscious attention, and sleep in humans. According to their results, in a task requiring sustained attention in blue-enriched white light compared to blue-free light, men had higher light perception and faster reaction time than women. The distribution of Psychomotor Vigilance Task (PVT) reaction times (the number of RT observations between 500-100 ms) illustrated that light at 6500 K color temperature led to the movement toward a faster RT range than light at 2500 K color temperature for men.

Sunlight seems to be the most effective among various light sources since it contains a sufficient amount and a wide range of light. Natural light, due to its role in the production of vitamin D in human blood, can improve mental mood, attention, cognitive function, physical activity, sleep quality, and consciousness ^52^. In their study, Shishegar et al^53^ analyzed the effects of daylighting on the health and consciousness of workers and students. As illustrated by their results, the health, satisfaction, attention, and performance of workers and students are improved by natural light^52^.

Furthermore, Sahin et al^54^ studies daylight exposure and its impacts on biomarkers, consciousness, and performance. They classified 13 subjects in illuminance ((low light (<5 lux), red light (max = 631 nm, 213 lux, 1.1W/m2), and white light (2568K, 361 lux, 1.1W /m2) conditions. The results of the mentioned study indicated that red light could increase the short-term performance, reduce reaction time significantly (P=0.05), and improve power in functional tests during a day. They also confirmed the hypothesis that exposure to daylight at long and narrow (red) or polychromatic wavelengths (2568 K) causes higher consciousness and shorter reaction time. The abovementioned results have been reported in similar studies performed by Figueiro et al.^55^ and Lafrance et al^56^.

Reaction time is the very short time that elapses between the presentation of a stimulus and the recording of the subject response. In healthy individuals, it usually lasts from 10-12 cent seconds, appearing voluntary and reflectively^57^. In other words, reaction time is the elapsed time for a person to understand the situation and process a response^58^. Smolders et al.^11^ have examined a mixed group of individuals (n=32) in different blocks through functional tests at two levels of illuminance (200lux or 1000lux at the eye level, 4000 K) for one hour in the morning and one hour in the afternoon. The results of the stated study showed that an increase in the illuminance (1000 lux, compared to 200lux) led to improved cognitive function, enhanced consciousness, less somnolence, more energy, and shorter reaction time.

Dehghan et al^59^indicated that after 90 min of exposure, simple, diagnostic, dichromatic selection, and two-tone selection reaction time were significant at all lighting levels (P<0.0001). According to the results of the referred study, after 90 min of exposure, the minimum and the maximum reaction time scores in illuminance were 200 lux and 1500 lux, respectively. In addition, the maximum and minimum response errors were at the level of 200 lux (0.5) and 500 lux (0.1), respectively. The removal response was also significant at different levels of illuminance (P=0.017); therefore, the maximum and minimum removal responses were reported at the level of 200 and 1500 lux (0.1), respectively. Chang (2013) has achieved similar results regarding the effects of light on attention and reaction time using the PVT test (psychomotor vigilance task) in people exposed to 1 lux illuminance, as compared to 90 lux^60^. Correa et al. have found that blue-enrich white light led to a greater improvement in reaction time with higher levels of baseline consciousness^61^.

"Attention" is a cognitive process defined as a selective focus on one aspect of the environment while ignoring others. It is also attributed to the allocation of resource processing^13^. The word "attention" can be defined in accordance with the number of errors made during a test; therefore, more assiduous attention during the test leads to fewer errors and vice versa^62^. An increase in attention function has been reported after 6.5 h of light exposure with short wavelengths (460 nm), compared to those with long wavelengths (550.55 nm)^63^. Dehghan et al^59^ investigated the effects of different levels of illuminance on the rate of attention and reaction time in laboratory conditions. The findings of the stated study indicated that the maximum and minimum percentages of attention in 1500 and 500 lux illuminance equal 99.75% and 99.36%, respectively.

This finding is in line with that obtained by Smolders et al^11^ who revealed that increasing illuminance improved cognitive functions in individuals. Amiri et al.^42^ also pointed out that although the mean scores of working memory and attention in low light level exposure are lower than harmless level exposure, this difference is not statistically significant. A field study conducted in schools has demonstrated that classroom lighting with variable illuminance ("focused" program: very bright, cold light: 1060 lux) enhanced attention in students^64^. The majority of studies reported that higher illuminance (1000-5000 lux vs. 5-200 lux for 1-5 h) is associated with increased function and attention^65,66^. Kretschmer suggested that exposure to bright light at night reduced error rates in tasks requiring memory and focused performance; nonetheless, it was is completely ineffective in tasks requiring sustained attention^33^.

## Conclusion

 As evidenced by the results of the present study, it can be stated that lighting affects the attention and reaction time; therefore, it should be designed to meet non-visual needs, apart from comfort and visual requirements. The parameters of the wavelength, color temperature, and intensity of light modulate the brain responses, including attention and reaction time. The best light in the regulation of psychological, biological, and cognitive processes is bright daylight in the morning with a short wavelength, high intensity, as well as stronger and more lasting effects. Shorter wavelengths, compared to the longer ones, lead to suppressed Melatonin, higher consciousness, less somnolence, increased attention function, and faster reaction time. When exposed to monochromatic light, non-visual responses are most sensitive to blue light (wavelengths between 459 and 483 nm).

**Figure 2:**
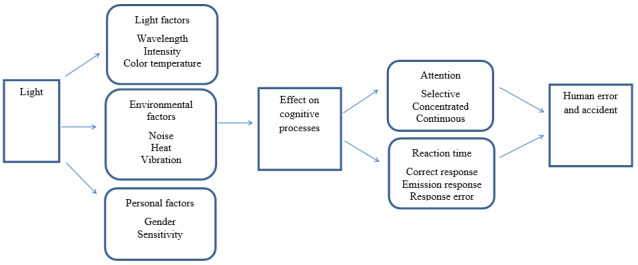
Conceptual model for the effects of light on attention and reaction time

The effects of blue light on the enhancement of cognitive function have been reported in various studies; nonetheless, direct exposure to it may cause retinal damage and oxidative stress. Illuminance, in addition to wavelength, affects reaction time and attention. The majority of studies have reported higher illuminance to be associated with increased consciousness, decreased somnolence, increased attention, and faster reaction time. Moreover, the assessment of the effects of color temperature demonstrated that higher color temperature is associated with greater melatonin suppression and faster reaction time in tasks that require attention. Therefore, the design and use of light in workplaces should be performed to meet non-visual and cognitive needs, such as attention and reaction time, in addition to providing comfort and visual needs. The effects of light on attention and reaction time can be presented through the following conceptual model. (Figure 2)

## Acknowledgments

 The authors would like to thank the Hamadan University of Medical Sciences for the library support of this study.

## Conflict of interests

 The authors declare that they have no conflict of interest.

## Funding

 Not funding.

 Highlights

- The light is a powerful modulator of non-visual performance in cognitive tasks.
- The light with shorter wavelengths, higher intensity, and higher color temperature lead to increased attention and faster reaction time.
- The best light in the regulation of psychological, biological, and cognitive processes is bright daylight in the morning with a short wavelength and high intensity.
- Simultaneous exposure to harmful levels of environmental factors interacts with cognitive and physiological parameters.
